# Utilization of social media peer support groups among family caregivers of patients with dementia

**DOI:** 10.1093/geroni/igab046.2947

**Published:** 2021-12-17

**Authors:** Mary Dioise Ramos

**Affiliations:** Kennesaw State University, Kennesaw, Georgia, United States

## Abstract

Caring for a family member with dementia is particularly challenging. Unpaid family caregivers provide a significant amount of the care for aging relatives, and they provide the vast majority of long-term care. Family caregiving often results in negative effects, which compromises the caregiver’s physical and psychosocial health. Social media support groups are an increasingly common venue for family caregivers supporting patients with dementia to exchange emotional, informational, and instrumental support. This study examined the utilization of social media support groups among family caregivers of patients with dementia during the pandemic. Using deductive thematic analysis, the use of social media support groups of family caregivers provide link to social interaction as a means when social distancing is enforced due to the pandemic. Family caregivers use social media support groups to share their personal experience, express their mood and feelings, offer prayers and positive quotes, keep up with the current events, gather information, and share feedback about dementia care services. Awareness of the potential advantages that social media support groups offer, healthcare providers can encourage family caregivers to use social media support groups as an empowering and practical platform. Further research is required about the long term benefit from social media support groups and the reliability and validity of the information that the family caregivers can get from the group.

